# Lower incidence of new onset depressive mood disorders associated with early postoperative weight-bearing versus non-weight bearing following fixation of femur and tibia fractures

**DOI:** 10.1007/s00402-025-06087-1

**Published:** 2025-10-14

**Authors:** Nicholas G. Belt, Andrew J. Moyal, Luc M. Fortier, Robert J. Burkhart, Jeremy M. Adelstein, Logan M. Good, Joshua K. Napora

**Affiliations:** 1 Case Western Reserve University, Cleveland, USA; 2 University Hospitals of Cleveland, Cleveland, USA

**Keywords:** Postoperative psychiatric outcomes, Lower extremity injuries, Intramedullary nailing, Open reduction and internal fixation, Weight-bearing status

## Abstract

**Introduction:**

Lower extremity fractures are common orthopaedic injuries and often require surgical intervention, which has been shown to significantly impact both the physical recovery and psychological well-being of patients. Given the high prevalence of psychiatric disorders following lower extremity injuries, understanding the relationship between postoperative weight bearing restrictions and psychiatric outcomes is important for improving patient recovery. It was hypothesized that patients with lower extremity fractures who were allowed early weight bearing postoperatively would have lower rates of new-onset psychiatric diagnoses.

**Methods:**

The TriNetX U.S Collaborative Network database was used to identify patients without prior psychiatric diagnoses who underwent either intramedullary nailing (IMN) or open reduction and internal fixation (ORIF) for femur and/or tibial fractures. The IMN and ORIF cohorts were matched based on age, sex, race, diabetes mellitus, and obesity. Psychiatric outcomes included depressive and anxiety disorders, substance use disorders, and psychotropic medication prescriptions. Outcomes were compared at early (1–7 days), intermediate (7 days-2 months), and long term (2 months-2 years) postoperative periods.

**Results:**

After matching, 7410 patients per cohort were included (IMN *n* = 7410; ORIF *n* = 7410; total *N* = 14,820). The rates of developing a new early (1–7 days) postoperative psychiatric diagnosis did not differ significantly between the IMN and ORIF cohorts. However, IMN was associated with a significantly lower rate of depressive mood disorders during the intermediate period (0.65% vs. 1.0%, *p* = 0.02) and over the two-year follow-up (3.1% vs. 3.7%, *p* = 0.03). After two years, IMN was associated with lower opioid use disorder compared to ORIF (0.20% vs. 0.50%, *p* = 0.012). There were no significant differences in the rates of anxiety disorders, substance use disorders, or psychotropic medication prescriptions at any timepoints.

**Conclusions:**

Early weight-bearing facilitated by IMN may be associated with a lower incidence of depressive mood disorders compared to delayed weight-bearing with ORIF. However, overall psychiatric outcomes were similar in this study. This suggests that other factors such as pain management and rehabilitation may play a more important role in postoperative psychiatric health.

## Introduction

Lower extremity injuries are among the most common and severe types of orthopedic injuries, accounting for 15% of emergency department visits in the US each year [1]. Lower extremity fractures often require surgical intervention such as intramedullary nailing (IMN) or open reduction and internal fixation (ORIF) [2–4]. When surgical intervention is required, the choice of surgical treatment and the subsequent weight-bearing protocol can significantly impact both the physical recovery and the psychological well-being of patients.

The negative psychological impact of orthopedic trauma and subsequent intervention is well documented. Numerous studies have indicated an increased prevalence of psychiatric disorders, such as depression [5, 6], anxiety [7, 8], and post-traumatic stress disorder [9, 10], following treatment. For instance, McCarthy et al. found that nearly half of patients with severe lower-limb injuries reported psychological distress at 3 months and 24 months post-injury [11]. Additionally, Wally et al. reported that patients with lower extremity orthopedic trauma had psychiatric disorder rates as high as 45% [12]. 

Given the high prevalence of psychiatric disorders following lower extremity injuries, understanding the relationship between the type of fracture management and psychiatric outcomes is important to improve the overall recovery of patients. This relationship, however, is not well characterized in the literature. This study aims to analyze the impact of lower extremity weight-bearing status on postoperative development of psychiatric disorders. By comparing patients treated with IMN versus ORIF for femur and/or tibial injuries, we hypothesize the early weight bearing status facilitated by IMN will be associated with better psychiatric outcomes compared to the delayed weight-bearing status that is necessitated by ORIF.

## Methods

The TriNetX U.S. Collaborative Network database is a health-research network.

comprising over 60 healthcare organizations with access to more than 116 million patients. The database is searchable using data such as International Classification of Diseases Tenth Revision (ICD-10) codes, Current Procedural Terminology (CPT) codes, demographic factors, Anatomical Therapeutic Chemical (ATC) codes, lab results, and vital signs. This study is exempt from Institutional Review Board (IRB) approval because the data used is a secondary analysis of existing data. It does not involve intervention or interaction with human subjects, and it is de-identified per the standard defined in the HIPAA Privacy Rule [13]. This study also followed the Strengthening the Reporting of Observational Studies in Epidemiology (STROBE) guidelines [13]. 

**Fig. 1:**
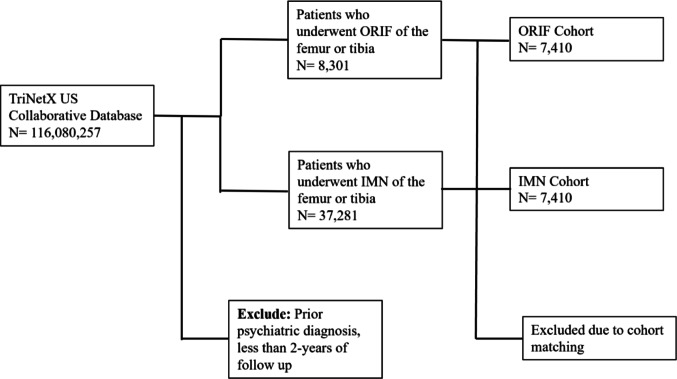
STOBE diagram depicting the patient selection process

The TriNetX database was queried on August 18, 2024 to identify patients who underwent IMN or ORIF procedures and did not have previous psychiatric diagnoses. For the ORIF cohort, CPT codes 27507 (open treatment of femoral shaft fracture with plate/screw, with or without cerclage) and 27758 (open treatment of tibial shaft fracture with plate/screws, with or without cerclage) were used to identify patients (Fig. 1). For the IMN cohort, CPT codes 27506 (open treatment of femoral shaft fracture, with or without external fixation, with insertion of intramedullary implant, with or without cerclage and/or locking screws) and 27759 (treatment of tibial shaft fracture by intramedullary implant, with or without interlocking screws and/or cerclage) were used to identify patients. For both cohorts, the exclusion criteria included any prior psychiatric pathology, which included ICD-10 codes F10-F19 (mental and behavioral disorders due to psychoactive substance use), F20-F29 (schizophrenia, schizotypal, delusional, and other non-mood psychotic disorders, F30-F39 (mood affective disorders), F40-F48 (anxiety, dissociative, stress-related, somatoform and other nonpsychotic mental disorders), F60-F69 (disorders of adult personality and behavior), and F99 (unspecified mental disorder). Additionally, patients were excluded from the IMN cohort if they had a concomitant ORIF procedure, and patients were excluded from the ORIF cohort if they had a concomitant IMN procedure. Since the TriNetX platform does not provide an injury‑severity index, patients with concomitant injuries (polytrauma) could not be systematically excluded or adjusted for. The cohorts were matched based on characteristics previously demonstrated to increase risk of psychiatric sequelae, including age, sex, race, diabetes mellitus, and obesity [9, 14–16]. There were 7,410 patients per cohort after propensity matching. The cohort characteristics are described in Table 1.

To assess the primary outcome of post-surgical psychiatric sequelae, patients that were diagnosed with depressive mood disorder, anxiety mood disorder, substance use disorder, opioid related disorder, or any psychiatric disorder (including all mood or substance use disorders) were identified using the following ICD-10 codes, respectively: F32-33, F41.1, F10-19, F11, and F01-98. Additionally, patients that were prescribed antidepressants and antipsychotics were identified using ATC codes N06A and N05A respectively. The rates of psychiatric diagnoses and prescriptions were compared between the two cohorts at an early postoperative period (1–7 days), an intermediate postoperative period (7 days-2 months), and a long-term postoperative period (2 months-2 years). The cohorts were compared using the statistical software available in the TriNetX platform. Categorical outcomes were compared using chi-squared analysis between the two different cohorts, while continuous outcomes were assessed with the Mann-Whitney U test. Statistical significance was defined by *p* < 0.05.

## Results

### Patient demographics

The final cohort of eligible patients consisted of 7410 patients in the ORIF cohort and 7410 patients in the IMN cohort, with a mean age of 48.4 and 48.5 years, respectively. (Table 1) Both cohorts were comprised of 51% male patients, of which 71% within each cohort identified as white. 9% of each cohort were diagnosed with diabetes at time of surgery, while 7% of the ORIF cohort and 6% of the IMN cohort was obese. (Table 1)

**Table 1:** Characteristics of the ORIF and IMN cohort after propensity matching

		ORIF (*n* = 7,410)	IMN (*n* = 7,410)
Age	Mean ± SD		
	Age at Index	48.4 ± 29.4	48.5 ± 29.4
Sex	n(%)		
	Male	3,793 (51)	3,751 (51)
	Female	3,206 (43)	3,234 (44)
	Unknown	411 (6)	425 (5)
Race	n(%)		
	White	5,229 (71)	5,229 (71)
	Black or African-American	732 (10)	727 (10)
	Hispanic or Latino	715 (10)	707 (10)
Diagnoses	n(%)		
	Diabetes Mellitus	691 (9)	648 (9)
	Obesity	528 (7)	480 (6)

*ORIF* open reduction internal fixation, *IMN* intramedullary nail

### Early postoperative period psychiatric diagnoses

**Fig. 2:**
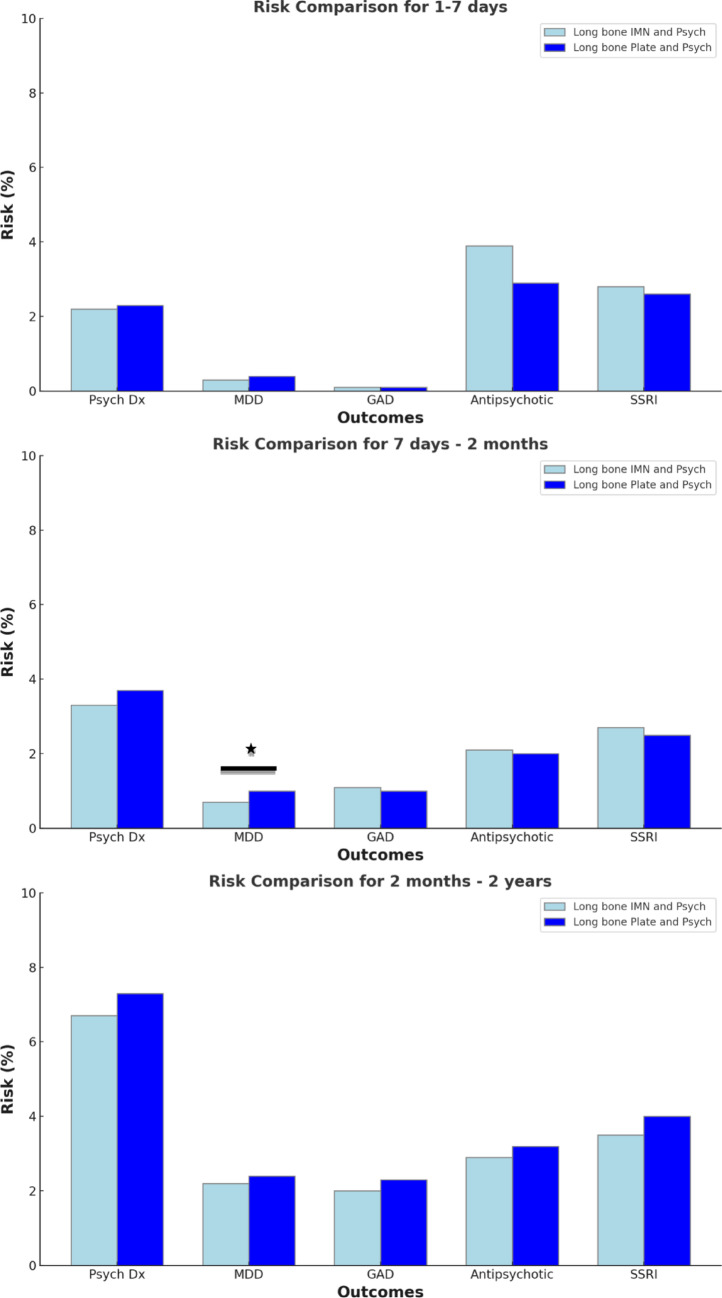
Bar graphs comparing certain outcomes following IMN versus ORIF at early-, intermediate-, and long-term postoperative periods. *ORIF* open reduction internal fixation, *IMN* intramedullary nail, *Psych Dx* psychiatric diagnosis, *MDD* major depressive disorder, *SSRI* selective serotonin reuptake inhibitors. * denotes statistical significance

During the early postoperative period (1–7 days), rates of psychiatric diagnoses did not differ between the two cohorts (2.2% IMN vs. 2.3% ORIF, *p* = 0.89) (Table 2; Fig. 2). The rate of antipsychotic prescription medication (3.9% IMN vs. 2.9% ORIF, *p* < 0.01) was higher in the IMN cohort. (Table 2; Fig. 2)

**Table 2:** Comparison of outcomes in the early (1–7 days) postoperative period

Outcomes	IMN	ORIF	Odds ratio	95% CI	*P*-value
Psychiatric diagnosis	2.20%	2.30%	0.98	(0.78–1.2)	0.89
Depressive mood disorders	0.30%	0.40%	0.78	(0.46–1.3)	0.35
Anxiety mood disorders	0.10%	0.10%	1	(0.42–2.4)	1
Substance use	0.60%	0.60%	1	(0.67–1.6)	0.91
Opioid	0.10%	0.10%	1	(0.42–2.4)	1
Meds	5.60%	4.90%	1.1	(0.96–1.3)	0.14
**Antipsychotic medication**	**3.90%**	**2.90%**	**1.4**	**(1.1–1.7)**	0.001*
Antidepressant medication	2.80%	2.60%	1.1	(0.86–1.3)	0.57

*ORIF* open reduction internal fixation, *IMN* intramedullary nail

* denotes statistical significance

### Intermediate postoperative period psychiatric diagnoses

During the intermediate postoperative period (7 days to 2 months), there were no differences between the cohorts with regards to rates of any psychiatric diagnosis (3.3% IMN vs. 3.7% ORIF; *p* = 0.21), anxiety mood disorders (1.1% IMN vs. 1.0% ORIF; *p* = 0.51), or substance use disorders (0.9% IMN vs. 1.0% ORIF; *p* = 0.27). (Table 3) The IMN cohort demonstrated a significantly lower rate of depression as compared to the ORIF cohort (0.7% IMN vs. 1.0% ORIF, *p* = 0.02). (Fig. 2) There was no difference in prescription rates for antidepressant medication (2.7% IMN vs. 2.5% ORIF; *p* = 0.54) or antipsychotic prescription (2.1% IMN vs. 2.0% ORIF, *p* = 0.93). (Table 3)

**Table 3:** Comparison of outcomes in the intermediate (7 days – 2 months) postoperative period

Outcomes	IMN	ORIF	Odds ratio	95% CI	*P*-value
Psychiatric diagnosis	3.30%	3.70%	0.89	(0.74–1.1)	0.21
Depressive mood disorders	0.70%	1.00%	0.66	(0.45–0.94)	0.022
Anxiety mood disorders	1.10%	1.00%	1.1	(0.81–1.5)	0.51
Substance use	0.90%	1.00%	0.83	(0.59–1.2)	0.27
Opioid	0.10%	0.10%	1	(0.42–2.4)	1
Meds	3.70%	3.70%	1	(0.82–1.2)	0.99
Antipsychotic medication	2.10%	2.00%	1	(0.78–1.3)	0.93
Antidepressant medication	2.70%	2.50%	1.1	(0.86–1.3)	0.54

*ORIF* open reduction internal fixation, *IMN* intramedullary nail

* denotes statistical significance

### Long-term postoperative period psychiatric diagnoses

During the long-term postoperative period (2 months to 2 years), the IMN and ORIF cohorts no longer statistically differed across any diagnosis or medication. (Table 4; Fig. 2)

**Table 4:** Comparison of outcomes in the long-term (2 months – 2 years) postoperative period

Outcomes	IMN	ORIF	Odds Ratio	95% CI	*P*-Value
Psychiatric diagnosis	6.70%	7.30%	0.91	(0.80–1.0)	0.2
Depressive mood disorders	2.20%	2.40%	0.91	(0.74–1.1)	0.42
Anxiety mood disorders	0.10%	0.10%	0.87	(0.70–1.1)	0.23
Substance use	2.10%	2.40%	0.9	(0.72–1.1)	0.35
Opioid	0.20%	0.30%	0.75	(0.38–1.5)	0.4
Meds	4.90%	5.50%	0.9	(0.75–1.1)	0.23
Antipsychotic medication	2.90%	3.20%	0.91	(0.73–1.1)	0.39
Antidepressant medication	3.50%	4.00%	0.86	(0.71–1.0)	0.1

*ORIF* open reduction internal fixation, *IMN* intramedullary nail

* denotes statistical significance

### Cumulative psychiatric diagnoses

**Fig. 3:**
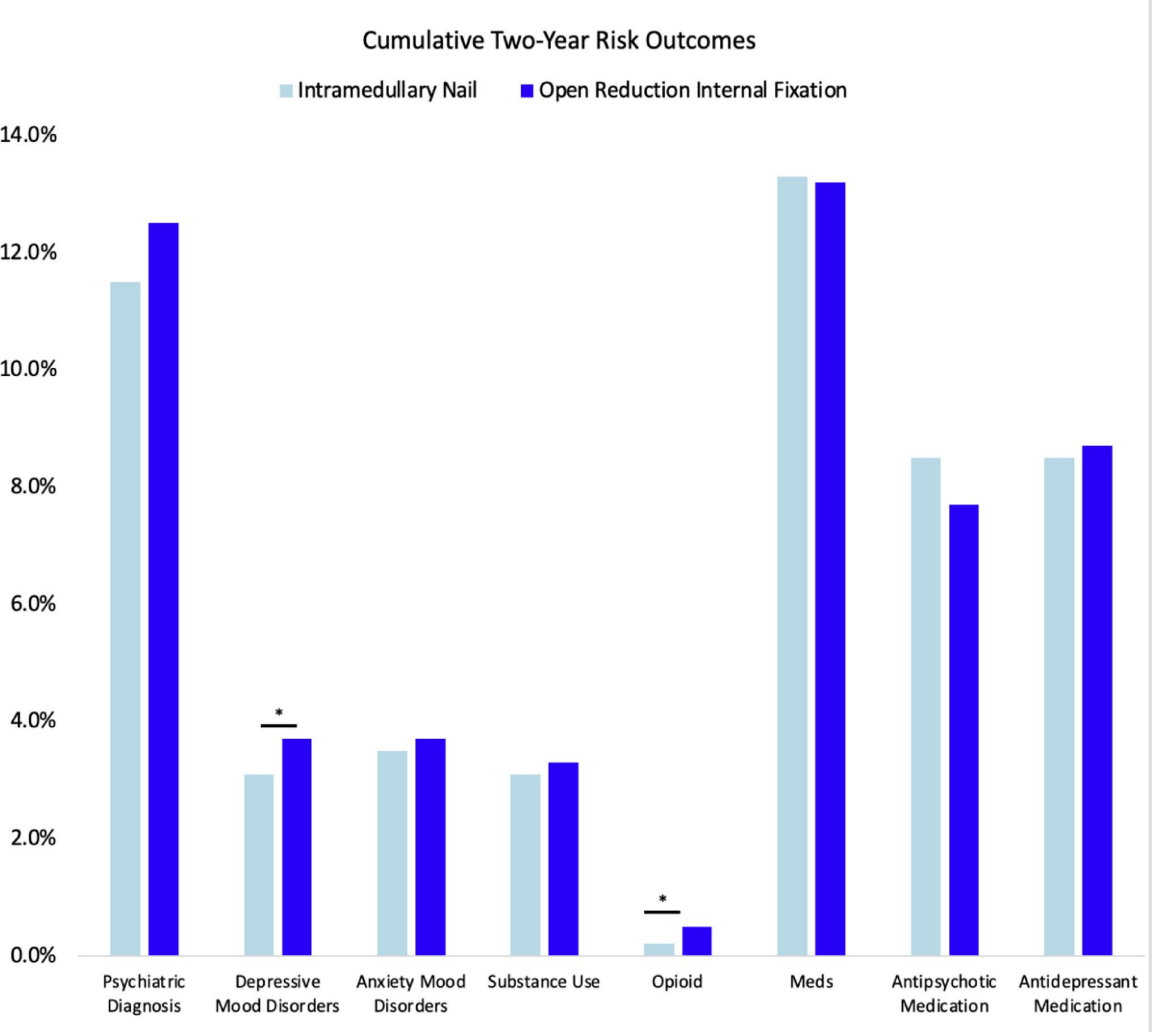
Bar graphs comparing certain cumulative two-year outcomes following IMN versus ORIF. *ORIF* open reduction internal fixation,*IMN* intramedullary nail. * denotes statistical significance

Across the entire 2-year follow up (0 days – 2 years), 12% of IMN and 13% of ORIF cohorts were diagnosed with a psychiatric condition (*p* = 0.1), differing only across a diagnosis of depression (3.1% IMN vs. 3.7% ORIF, *p* = 0.03) and opiate use disorder (0.20% IMN vs. 0.50% ORIF, *p* = 0.01). (Table 5; Fig. 3) There were similar percentages of antidepressant use (8.5% IMN vs. 8.7 ORIF, *p* = 0.7) and antipsychotic use (8.5% IMN vs. 7.7% ORIF, *p* = 0.11) across cohorts.

**Table 5:** Comparison of outcomes cumulatively from 1 day – 2 years postoperatively

Outcomes	IMN	ORIF	Odds Ratio	95% CI	*P*-Value
Psychiatric diagnosis	12%	13%	0.92	(0.84–1.0)	0.1
Generalized anxiety disorder	3.50%	3.70%	0.95	(0.80–1.1)	0.6
**Major depressive disorder**	**3.10%**	**3.70%**	**0.82**	**(0.69–0.99)**	0.033*
Bipolar Disorder	0.20%	0.10%	1.4	(0.63–3.0)	0.43
Substance Use	3.10%	3.30%	0.92	(0.77–1.1)	0.37
**Opioid use**	**0.20%**	**0.50%**	**0.49**	**(0.27–0.87)**	0.012*
Alcohol use	0.70%	0.80%	0.86	(0.59–1.3)	0.44
Cannabis use	0.30%	0.40%	0.93	(0.54–1.6)	0.78
Antidepressant medication	8.50%	8.70%	0.98	(0.86–1.1)	0.7
Antipsychotic medication	8.50%	7.70%	1.1	(0.98–1.3)	0.11
Suicide	0.10%	0.10%	1	(0.42–2.4)	1

*ORIF* open reduction internal fixation, *IMN* intramedullary nail

* denotes statistical significance

## Discussion

This study found that there was no significant difference in the overall psychiatric outcomes between the IMN (early-weight bearing) and ORIF (delayed-weight bearing) cohorts. However, the IMN group had a significantly lower rate of depressive mood disorders during the intermediate postoperative period and across the 2-year follow up. Additionally, across the two‑year follow‑up, patients treated with IMN had a significantly lower rate of opioid use disorder than those treated with ORIF. Despite these findings, the rates of any psychiatric diagnoses, anxiety disorders, and prescriptions for antipsychotic and antidepressant medications were similar between the two groups. This suggests that while early weight bearing may have some benefit in reducing depressive mood disorders, it does not appear to have a significant impact on other psychiatric outcomes or prescription medication use.

Our results do partially support the hypothesis that early weight bearing facilitated by IMN is associated with better psychological outcomes, specifically in terms of lower rates of depression. This aligns with existing literature that highlights the benefits of physical activity on psychological well-being [17–21]. There have also been studies that show physical inactivity is associated with increased risk of anxiety and depressive disorders [17, 22]. These findings are also consistent with findings from a recent randomized controlled trial (RCT) by Dehghan et al. in which patients undergoing ankle ORIF were randomized to an early weightbearing versus non-weightbearing protocol [23]. The results demonstrated a statistically significant better SF-36 mental health score (66 vs. 54, *p* = 0.0008) in the early weight bearing cohort as compared to the non-weightbearing cohort at 6 weeks postoperatively. Similarly, Stassen et al.’s RCT comparing early weight bearing and non-weight bearing protocols following isolated stable Weber B ankle fractures found significantly improved RAND mental scores (78.5 vs. 58.2, *p* = 0.034) in the early weight bearing cohort [24]. Another recent prospective study by Kalmet et al. compared outcomes from permissive weight bearing to restricted weight bearing in surgically treated trauma patients with peri- and intra-articular fractures of the lower extremities [25]. The results demonstrated significantly better subjective activities of daily living (ADL) and quality of life in the permissive weight bearing group compared to the restricted weight bearing group, which further strengthens the findings from our study that restricted weight bearing is associated with increased rates of depression. A previous systematic review of nine studies by Black et al. sought to evaluate the advantages of early weight bearing following ankle fractures [26]. The authors concluded that there is evidence to suggest early weight-bearing may allow for earlier return to work [27] and shorter hospital stays [28], which may be a contributing factor to the reduction in rates of depression identified in our study. Longer hospital length of stay in postoperative fracture patients with restricted weight bearing was also observed in a scoping review performed by Aloraibi et al. [29]. It is well documented that the length of time spent in the hospital has negative effects on mental health [30–32]. 

While early mobility is believed to offer benefits, the absence of significant differences in psychiatric outcomes beyond depression and opioid use disorder between the IMN and ORIF groups suggests that other factors may have a greater influence on postoperative psychiatric health. Previous psychiatric diagnoses are a well-documented risk factor for postoperative psychiatric conditions, and excluding these patients from our study likely contributed to the lower overall rates of psychiatric diagnoses observed [33]. Additionally, factors such as pain management and rehabilitation difficulties have been identified as significant contributors to psychiatric sequelae following orthopedic trauma [33]. 

This study is not without limitations. The retrospective nature of the study and reliance on the TriNetX database limit the availability of detailed patient-specific information. Specifically, the lack of explicit information regarding postoperative weight bearing status required this study to utilize surgical fixation type (IMN vs. ORIF) as a proxy for weight bearing status, which relies on making an assumption that may not always be accurate. We were also unable to account for the presence and severity of concomitant injuries. Polytrauma may necessitate delayed weight‑bearing, prolonged immobilization, and longer hospitalizations—factors associated with worse mental‑health outcomes—potentially confounding observed differences between IMN and ORIF. Additionally, the exclusion of patients with previous psychiatric diagnoses, while necessary for isolating the impact of weight-bearing status, may have reduced the generalizability of our findings. The matching process, although rigorous, cannot account for all potential confounding variables. Furthermore, the potential confounding impact of pain management and rehabilitation efforts on psychiatric outcomes was not able to be parsed out due to limits of TriNetX’s database. Future prospective studies should explore these variables in more detail and consider longer follow-up periods to better understand the relationship between weight-bearing status and psychiatric health. Investigating the roles of pain management, rehabilitation efforts, weight-bearing status, and other psychosocial factors will be crucial in identifying patients at risk of developing psychiatric illnesses following orthopedic trauma. Additionally, exploring the impact of different types and intensities of physical activity postoperatively on psychiatric outcomes could provide more detailed insights into optimizing recovery for orthopedic trauma patients.

## Conclusion

Our study demonstrates that early weight-bearing facilitated by IMN is associated with a lower rate of depressive mood disorders during the intermediate postoperative period and across the 2-year follow-up period compared to delayed weight-bearing with ORIF. However, the overall psychiatric outcomes were similar between the two groups, suggesting that other factors, such as pain management and rehabilitation, play a more significant role in postoperative psychiatric health. Physicians should consider these factors when developing postoperative care plans to identify and manage patients at risk of developing depression following surgery.

## Data Availability

No datasets were generated or analysed during the current study.
